# Treatment of a middle cerebral artery bifurcation aneurysm with the novel Contour Neurovascular System compatible with 0.021″ catheters

**DOI:** 10.1177/19714009211041523

**Published:** 2021-08-23

**Authors:** Maximilian Thormann, Anastasios Mpotsaris, Daniel Behme

**Affiliations:** University Clinic for Neuroradiology, University Clinic Magdeburg, Germany

**Keywords:** Intracranial aneurysm, flow diversion, contour device

## Abstract

**Background:**

For wide-necked intracranial aneurysms, endo-saccular flow disruption can be a viable alternative to coiling or flow diverters. The Contour Neurovascular System is an intrasaccular flow diverter device targeting the neck of the aneurysm. Until now, the system had to be delivered through a 0.027″ microcatheter. We report the first implantation and follow-up of the novel Contour 021 system compatible with 0.021″ microcatheters.

**Case presentation:** A 54-year-old male patient presented with an unruptured right middle cerebral artery aneurysm at the right temporopolar branch. Existing medication included apixaban. An arteriogram showed a broad-based aneurysm. Due to its asymmetric geometry, neither the Woven EndoBridge nor stent-assisted coil embolisation were regarded as promising treatment strategies. To uphold the option of different treatment options, prasugrel 10 mg was initiated before treatment. Implantation was performed under general anaesthesia via femoral artery puncture. A 0.021″ Headway™ catheter was used for accessing the aneurysm. The Contour device was oversized to the equatorial plane. Deployment was successful with only one attempt without the need for re-sheathing. Follow-up catheter angiography was performed after three months, showing complete occlusion of the aneurysm. No procedure-related complications occurred.

**Conclusion:**

The 0.021 design of the Contour enlarges the subgroup of patients that can be treated with endo-saccular devices and will enable treatment of smaller and more distal aneurysms.

## Introduction

**Figure 1.**
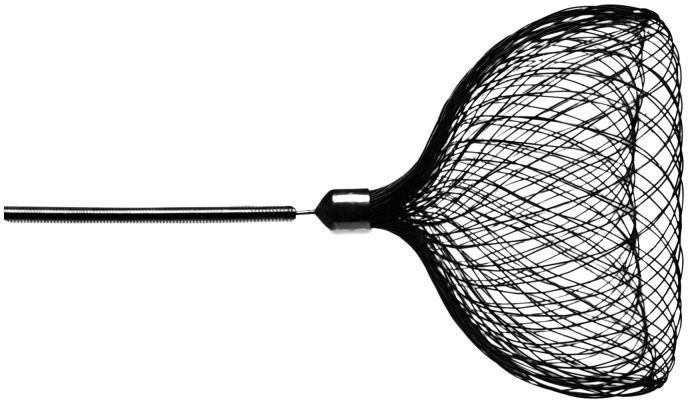
Deployed configuration of the Contour Neurovascular System.

Endo-saccular flow disruption is an emerging strategy for the treatment of wide-necked intracranial aneurysms. In contrast to endoluminal flow diversion, it has the advantage that dual antiplatelet therapy is not necessary.^1^ Furthermore, coverage of side branches is avoided. Especially for wide-necked aneurysms, endo-saccular devices can be a viable alternative to coiling or flow diverters.^2^ The Contour Neurovascular System (Cerus Endovascular, Fremont, CA) is an intrasaccular flow diverter system targeting the neck of the aneurysm. It consists of a Nitinol micro-braided mesh available in a range of sizes applicable to both small and medium-sized aneurysms (Figure 1). In its deployed configuration, the device adapts to the contours of the aneurysm and takes a chalice or cup-like form. Its effect consists of a combination of flow diversion at the neck by reconstructing the bifurcation and flow disruption within the aneurysm, avoiding manipulation of the fragile dome.^2^ However, until now, the system had to be delivered through a 0.027″ microcatheter, which limited its usability in anatomically challenging cases.

We present the first-in-human implantation and follow-up of the novel Contour 021 system compatible with 0.021″ microcatheters.

## Case

**Figure 2.**
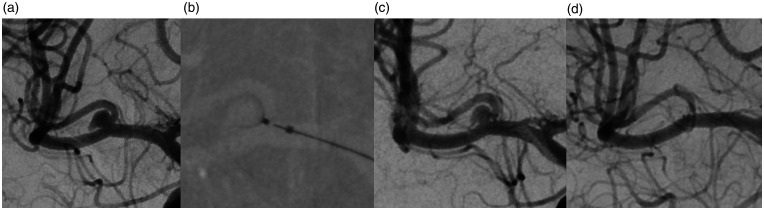
Digital subtraction angiography (DSA) anterior posterior view of the right internal carotid artery (a) shows an unruptured middle cerebral artery aneurysm at the right temporopolar branch. DSA at deployment of the 021 Contour device (b) and three minutes after deployment (c). Three-month follow-up shows complete occlusion of the aneurysm (d).

The patient was a 54-year-old male presenting with an incidental unruptured right middle cerebral artery (MCA) aneurysm at the right temporopolar branch. In an anatomical variant, the latter branches off cranially at an acute angle. He had no known prior neurological deficit. Due to a prothrombotic syndrome, the patient was taking apixaban. On the initial angiogram, the neck of the aneurysm was broad based, and the dome carried a small lateral daughter sac. The anterior posterior (AP) diameter was 5 mm, the dome 5.5 mm and the neck diameter 2.5 mm (Figure 2). The case was discussed in an interdisciplinary neurovascular team meeting with the neurosurgery and neurology departments, and the aneurysm was considered suitable for endovascular treatment. Because of the asymmetric situation at the MCA bifurcation, we felt neither the Woven EndoBridge (WEB; Microvention, Aliso Viejo, CA) nor stent-assisted coil embolisation to be promising strategies. After discussing different treatment options with the patient, he agreed on treatment with the Contour Neurovascular System (Cerus Endovascular). To ensure different treatment options would still be possible (stent/flow diversion), we decided to medicate with prasugrel 10 mg starting the day before the treatment in addition to apixaban.

### Procedural technique

The case was performed by A.M. under general anaesthesia via standard common femoral artery puncture and an 8Fr sheath insertion. A 6Fr Cerebase (Cerenovus, Miami, FL) guide sheath was navigated into the target main artery. A Sofia 5Fr (Microvention) was used as a distal access catheter. After diagnostic runs, 3D digital subtraction angiography was performed, and working projections were chosen accordingly.^3^ A 0.021″ Headway™ catheter (Microvention) and a 0.014″ Synchro microwire (Stryker, Kalamazoo, MI) were used for catheterisation of the aneurysm. The device was oversized to the equatorial plane of the aneurysm, as reported previously.^2^ The device was implanted with only one attempt without the need for re-sheathing. After deployment, post-procedural (immediate and delayed) angiographic runs were performed to assess device placement and for flow/stasis within the aneurysm and to monitor for any immediate complications. We did not perform a post-procedural flat panel computed tomography scan. Follow-up catheter angiography was performed after three months showing complete occlusion of the aneurysm (Figure 2). After the follow-up angiogram, prasugrel treatment was stopped. No neurological deficit occurred after the intervention or up until now.

## Discussion

To the best of our knowledge, we present the first-in-human implantation and follow-up of the novel Contour Neurovascular System 021 device (Cerus Endovascular). Implantation of the device was technically feasible, and three-month results showed no sign of device movement or compression and full stasis within the aneurysm.

While effective coiling for complex aneurysm geometry is often technically challenging, numerous new devices have been developed, including endo-saccular and endoluminal technologies.^4^ Flow diverters have shown to be an effective treatment in particular for side-wall aneurysms. Yet, problems may arise with bifurcation aneurysms and occlusion of side branches.^5^ Endo-saccular flow disruption devices such as the WEB (Microvention) or the Contour device are deployed within the aneurysm, intending to create a doubling effect with intra-aneurysmal flow disruption and induction of thrombosis followed by a remodelling of the parent artery.^1^ The mesh positioned at the aneurysm neck can lead to neo-endothelial overgrowth, making them well suited for wide-necked bifurcation aneurysms.^4^ However, the WEB has a symmetric geometry which makes it difficult to be implanted in a perfect position in some asymmetric aneurysm geometries.^6^

The 027 Contour device is one of the more recent additions to the market of endo-saccular flow disruption devices. Clinical reports have shown safe and effective implantation.^1,2^ It is available in four sizes. Sizing is based on the widest diameter of the neck using a predefined table. Up to now, a 0.027″ microcatheter was needed for device deployment. Due it its shape, the Contour has properties of both flow diversion and flow disruption. A small series of 11 patients treated with the 027 design showed complete occlusion rates of 55.6% after one year, with all patients showing Raymond Roy Class 1 or 2.^2^ Other than with purely endoluminal flow diversion, the Contour device has no parent vessel component, and continuous anti-platelet therapy is not needed.

The new 021 design allows for treatment of smaller aneurysms sizes usually not accessible with larger microcatheters. Tortuosity of vessels may make navigating to the aneurysm base with an 0.027″ catheter challenging if not impossible.^2^ The smaller catheter size provides more flexibility in overcoming geometrical issues. There were no problems accessing the aneurysm base in our case, and implantation succeeded at first attempt. The Contour fully opened at deployment. Sizing according to the provided table was uncomplicated and correct.

The 021 design of the Contour enlarges the subgroup of patients who can be successfully treated with endo-saccular devices and who can benefit from endovascular treatment of aneurysms. The smaller catheter size will enable interventionalists to treat more distally located aneurysms. Evaluation of efficacy and safety of the device will require larger series and longer follow-up times.

## Conclusion

The Contour Neurovascular System 021 device is a promising development enabling to offer treatment to patients with aneurysms not suitable for treatment with larger microcatheters or other devices.
